# Investigation into the subjective well-being of older adults via confirmatory factor analysis and analytic hierarchy process—an empirical analysis based on CGSS2021 data

**DOI:** 10.3389/fpubh.2025.1585408

**Published:** 2025-08-19

**Authors:** Yingying Chen, Tianrui Cai, Ruiqin Tian

**Affiliations:** School of Mathematics, Hangzhou Normal University, Hangzhou, China

**Keywords:** older adults, subjective well-being, information entropy method, confirmatory factor analysis, analytic hierarchy process

## Abstract

**Background:**

This paper examines the subjective well-being of older adults in China. Based on data from the China General Social Survey (CGSS2021), the agreement levels of 21 questions on the subjective well-being of older adults were extracted and analyzed.

**Methods:**

The 21 questions were categorized into positive and negative factors based on cluster analysis. For the 10 positive factors, the information entropy method was used to determine their weights and calculate the happiness scores of older adults. For the 11 negative factors, the unhappiness scores of older adults were calculated by the confirmatory factor analysis and analytic hierarchy process.

**Results:**

An average happiness score of 4.499 was obtained, of which the more influential factors were family income, personality control, and the natural environment. An average score of 3.062 was obtained, among them, the more influential factors are health status, family communication and body parts.

**Conclusions and recommendations:**

Overall, it shows that the subjective happiness of the Chinese older adults is strong and unhappiness is weak. Then we further find significant differences in the above scores of older adults with different socioeconomic status through one-way ANOVA. Based on the results of the study, we finally proposed targeted recommendations to improve the happiness feelings of older adults, including encouraging communication with family members and improving body parts.

## 1 Introduction

According to the seventh national census, older adults demographic, those aged 60 and above (1), constitutes 18.7% of China’s population (2), with those over 65 accounting for 13.5%, indicating a significantly aging populace (3). Projections for 2050 foresee this cohort rising to 34.91%. Amidst relentless societal progress, this demographic shift results in diminished mobility among older adults and psychological challenges such as loneliness, anxiety, and depression, impacting their subjective well-being and quality of life. A comprehensive analysis of influential factors is imperative for enhancing their well-being. This study employs confirmatory factor analysis and analytic hierarchy process to examine data from the CGSS2021, aiming to provide theoretical support for improving older adults’ mental health.

Subjective Well-Being (SWB) is a multidimensional construct reflecting individuals’ evaluations and emotional responses to their life experiences, encompassing cognitive and affective dimensions (4). The cognitive facet pertains to life satisfaction, while the affective element involves positive and negative emotional experiences. Research indicates a U-shaped correlation between subjective well-being and age, with variations observed post-70s in Europe and Central Asia (5). A holistic comprehension requires considering psychological attributes, sociocultural contexts, and lifecycle dynamics. Understanding older adults subjective well-being is pivotal for strategies to sustain their health, foster familial concord, and societal equilibrium, and bolster economic prosperity (6).

## 2 Literature review

1. *Educational attainment*: this is a pivotal determinant of the subjective well-being among older adults. The research underscores that education enriches not only one’s cognitive reserves but also significantly influences life quality and psychological satisfaction in later life by bolstering self-identity and self-esteem. Huang (7) elucidates that individuals with advanced educational backgrounds, including high school, junior college, and university degrees, exhibit heightened subjective well-being. Jin et al. (8) accentuated education’s role in the well-being of older adults, with higher educational attainment linked to superior health awareness and broader social resource access, which are conducive to the well-being of older adults.
2. *Social support*: Yao et al. (9) study, published in the Psychological Journal, provided an insightful exploration into the psychological impact of social support on the social well-being of older adults and the mechanisms at play. The research revealed that social support exerts a substantial influence on social well-being by fostering hope and mitigating feelings of loneliness. Jia and Zhou (10) underscored the positive role of social support, encompassing familial bonds and social networks, in enhancing psychological well-being and life satisfaction among older adults. It was observed that increased social support correlates with diminished loneliness and elevated life satisfaction and subjective well-being among older adults.
3. *Socioeconomic status*: Wang (11) study meticulously examined the mental health implications of socioeconomic status among older adults, uncovering a robust positive correlation with their mental well-being. It was observed that elevated income levels, higher social strata, and greater self-assessed socioeconomic standing are indicative of the older adults’ enhanced subjective well-being, a conclusion that withstood the test of statistical scrutiny. Furthermore, the study highlighted that augmenting perceptions of fairness, broadening social interactions, and refining leisure and recreational activities are pivotal conduits through which socioeconomic status positively influences the subjective well-being of older adults.
4. *Residential milieu*: Guo (12) highlighted that the residential milieu and social engagement significantly influence the psychological well-being of older adults. An auspicious living environment can furnish a congenial habitat and augment life contentment, whereas social engagement can mitigate solitude, amplify the perception of social integration, and exert a salutary influence on the subjective well-being of seniors.
5. *Self-health assessment*: Yin et al. (13) have elucidated that health self-assessment is intricately linked to the subjective well-being of older adults. A favorable health self-assessment is correlated with heightened life satisfaction and diminished depression among older adults, consequently bolstering their well-being. Cai et al. (14) have underscored that the health status of older adults is a direct determinant of their subjective well-being. Older adults in good health are more inclined to report elevated levels of well-being. Moreover, health issues and functional impairments may curtail older adults’ engagement in social activities, thereby impacting their well-being, and depressive symptoms exert a deleterious effect on the well-being of older adults.
6. *Internet utilization*: Ding et al. (15) have underscored the profound influence of internet utilization on the subjective well-being of older adults. Engaging in online learning, social interactions, and recreational pursuits, internet usage augments older adults’ perception of social engagement and autonomy, thus elevating their overall well-being and sense of community. Conversely, Kraut et al. (16) have determined that excessive digital immersion can lead to diminished social involvement, curtailed communication with kin, and a constricted social network, potentially intensifying sentiments of melancholy and solitude, and diminishing the well-being of older adults.
7. *Marital status*: Shi et al. (17) have highlighted that marital status exerts a substantial influence on the well-being of older individuals. Unmarried or widowed seniors are prone to encounter diminished well-being. Matrimony offers avenues for emotional succor and social communion, which are pivotal to the well-being of the aging population.
8. *Scholarly interest in seniors’ subjective well-being:* Yu et al. (18) research indicates that the contemporary state and determinants of subjective well-being among older adults garnered scholarly attention in the 1950s, with studies on positive influences on subjective well-being progressively surfacing, spurred by the advent of the positive psychology movement.
9. *Variations in older adults subjective well-being*: The subjective well-being of older adults is subject to individual differences, yet broadly, numerous seniors experience a comparatively elevated quality of life and well-being. This is attributable to an enhanced old-age welfare system, expanded avenues for social engagement and interaction, superior health status and medical care, and the presence of agreeable living environments (19). Broadly, a rising trend in subjective well-being is observed among older adults in numerous nations, albeit with a minority of countries exhibiting reduced well-being levels. Nonetheless, certain senior individuals confront challenges such as social seclusion, economic hardships, health complications, and other factors that impinge upon their subjective well-being.

## 3 Data sources

This study utilizes data from the CGSS2021, conducted by the Institute of Sociology at the Chinese Academy of Social Sciences (CASS) in 2021. The survey employed probability sampling for household interviews, covering 21 regions with a sample size of 8,148 individuals. After data screening and imputing missing values, we analyzed individuals aged 50 and above, resulting in a sample size of 1,223. The demographic details of this cohort are presented in Table 1.

**Table 1:** Basic information of the samples.

Category	Information	Number of samples	Percentage (%)
Gender	Male	572	46.77
	Female	651	53.23
Age	51–60	445	36.39
	61–70	406	33.20
	71–80	297	24.28
	81+	75	6.13
Education level	Primary and below	489	39.98
	Junior School	408	33.36
	High School, technical, vocational	229	18.72
	College and above	97	7.93
Health status	Very unhealthy	87	7.11
	Unhealthy	221	18.07
	Average	399	32.62
	Healthy	369	30.17
	Very healthy	147	12.02
Socioeconomic status	Upper	10	0.82
	Upper-middle	74	6.05
	Middle	455	37.20
	Lower-middle	370	30.25
	Lower	314	25.67

## 4 Study design and results

To facilitate subsequent modeling, we extracted relevant phrases from the D35 form within the questionnaire, initially categorizing them as positive (P) or negative (N) based on their semantic content. The findings are presented in Table 2.

**Table 2:** Refinement of the results of the questionnaire.

Original item	Refined item	Valence
Social opportunities are increasing	Social resources	P
As I age, I’ve learned many life lessons, making me stronger and more capable	Mental toughness	P
Most of the life goals I set encourage me rather than discourage	Goal encouragement	P
I often feel like I’m just marking time	Time management	N
I’m not clear on the meaning of what I’ve done in my life	Life value	N
I often feel discomfort in certain parts of my body	Body parts	N
Compared to those around me, I am content	Satisfaction in life	P
I am satisfied with my family’s income	Family income	P
I often worry about trivial matters	Trivial things	N
I am often troubled by my health condition	Health status	N
I find it hard to form friendships with others	Friendship establishment	N
I like my personality	Personality control	P
I feel like most people have more friends than me	Number of friends	N
Being with family makes me especially happy	Family relationship	P
I feel like I have worse luck than others	Degree of luck	N
I am confident in society’s development	Social outlook	P
Compared to those around me, I feel at a disadvantage	Degree of loss	N
When encountering unhappy events, it takes me a long time to regain my spirits	Emotional regulation	N
I am pleased that my views have matured over the years	Subjective thoughts	P
I sometimes find it difficult to communicate with family members	Family communication	N
I am satisfied with the natural environment around me	Natural environment	P

To enhance the forthcoming modeling, we eliminated the responses of “do not know” and “refusal to answer.” Subsequently, we apportioned a numerical value to the level of concordance for each of the 21 tabled queries, sequentially graded as follows: Strongly Disagree = 1; Disagree = 2; Slightly Disagree = 3; Slightly Agree = 4; Agree = 5; Strongly Agree = 6.

In this study, each variable is clearly defined based on relevant psychological theories. Social resources, based on social support theory, refer to accessible and perceivable social networks, community resources, and participation opportunities, crucial for mental health and well-being. Mental toughness, based on psychological resilience theory, refers to the ability to recover from hardships, manage stress, and achieve positive growth. Goal encouragement, based on goal - setting theory, represents the motivating power of self - defined goals, spurring action and self - efficacy.

Time management, based on self - regulation theory, involves time planning, distribution, and oversight to boost efficiency and balance life aspects. Life value, in humanistic psychology, is the subjective understanding of existence meaning, life purpose, and activity worth. Body parts, based on mind - body interaction theory, refer to subjective awareness of physical sensations and health conditions, impacting emotions.

Satisfaction in life, based on subjective well-being theory, is cognitive and emotional contentment with life quality. Family income, in economic psychology, includes family earnings and individuals’ financial satisfaction. Trivial things, based on emotion psychology, are daily nuisances evoking negative feelings. Health status, in health psychology, is the subjective appraisal of physical health and disease influence.

Friendship establishment, based on social interaction theory, is building intimate connections through social communication. Personality control, based on personality psychology, is the ability to identify, embrace, and regulate traits for social adaptation. Number of friends, in social network theory, reflects social connection scale and belonging. Family relationship, in family systems theory, focuses on emotional and behavioral interaction quality among family members.

Degree of luck, based on cognitive psychology, is the subjective judgment of life event favorability. Social outlook, in social - cognitive theory, is anticipation and confidence in social development trends. Degree of loss, based on social comparison theory, is perceived disadvantage in resource and benefit comparison. Emotional regulation, based on emotion regulation theory, is applying strategies to manage emotions and maintain equilibrium.

Subjective thoughts, in cognitive development theory, are distinctive cognitions formed through personal experiences. Family communication, based on communication theory, is information and emotion exchange within the family. Natural environment, in environmental psychology, is the subjective assessment of natural ecological and landscape elements.

### 4.1 Descriptive statistics of the variables

To obtain a comprehensive overview of the responses to each query, we calculated the mean and standard deviation of the agreement levels for the 21 questions, with the results outlined in Table 3.

**Table 3:** Descriptive statistics of the variables.

Question number	Variable names	Average value	Standard deviation
D35_1	Social resources	4.74	0.946
D35_2	Mental toughness	4.84	0.807
D35_3	Goal encouragement	4.66	0.873
D35_4	Time management	2.85	1.352
D35_5	Life value	2.84	1.340
D35_6	Body parts	3.50	1.569
D35_7	Satisfaction in life	4.64	1.046
D35_8	Family income	3.95	1.369
D35_9	Trivial things	3.03	1.398
D35_10	Health status	3.10	1.520
D35_11	Friendship establishment	2.67	1.298
D35_12	Personality control	4.47	1.114
D35_13	Number of friends	3.25	1.384
D35_14	Family relationship	5.07	0.787
D35_15	Degree of luck	2.95	1.391
D35_16	Social outlook	5.00	0.902
D35_17	Degree of loss	2.87	1.355
D35_18	Emotional regulation	2.95	1.424
D35_19	Subjective thoughts	4.72	0.922
D35_20	Family communication	2.96	1.446
D35_21	Natural environment	4.62	1.100

The scoring criteria for agreement are as follows: “Strongly agree” is assigned a score of 6, “Agree” a score of 5, “Somewhat agree” a score of 4, “Somewhat disagree” a score of 3, “Disagree” a score of 2, and “Strongly disagree” a score of 1. Data analysis reveals a significant disparity in the mean scores between questions indicating positive sentiments (1, 2, 3, 7, 8, 12, 14, 16, 19, 21) and those indicating negative sentiments (4, 5, 6, 9, 10, 11, 13, 15, 17, 18, 20). The standard deviation of the agreement scores for the 21 questions is approximately 1, indicating a high degree of consistency in older adults’ level of agreement with each question.

### 4.2 Analysis of systematic clustering results

**Figure 1:**
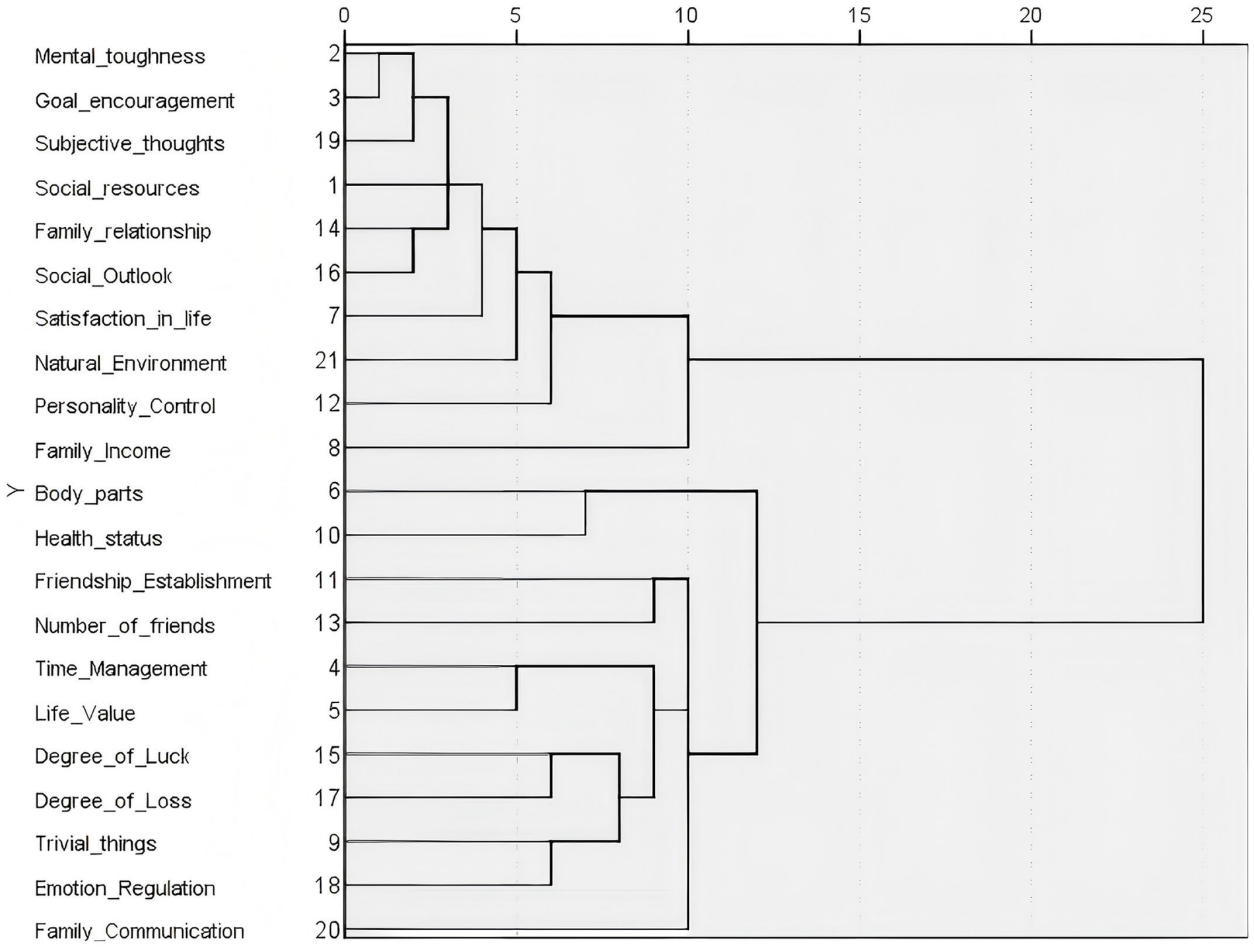
Dendrogram of clustering results.

These 21 indicators were subjected to systematic clustering analysis using SPSS, with the dendrogram of the clustering outcomes presented in Figure 1 (20).

As illustrated in the figure, the significant divergence in mean values between questions reflecting positive and negative sentiments led to their segregation into two distinct clusters. The outcomes of the cluster analysis align perfectly with our initial semantic assessments. Among the 10 questions indicative of positive sentiments, further stratification was found to be infeasible. Subsequent confirmatory factor analysis, despite numerous refinements to the model’s path diagram, yielded unsatisfactory fitting indices, leading us to conclude that these 10 questions are not amenable to confirmatory factor analysis. Consequently, we employed the information entropy method to ascertain the weights of the 10 questions, enabling the computation of well-being scores for each older adult individual, reflecting positive sentiments, on a weighted basis.

The 11 questions reflecting negative sentiments exhibit a clear hierarchical structure. Based on the clustering results and the specific meanings of each factor, the secondary indicators of the agreeableness evaluation index factors can be categorized into four groups: physical health, encompassing body parts and health status questions; interpersonal relationships, including friendship establishment, the number of friends, and family communication; value management, with questions on time management and life value; and emotional control, comprising the degree of luck, the degree of loss, trivial matters, and emotional regulation.

### 4.3 Modeling of positive factors based on the information entropy method

#### 4.3.1 Determine the weight of each index of positive factors based on the information entropy method

The information entropy method allows us to assess the degree of dispersion for a certain indicator. According to the definition of information entropy, a smaller entropy value indicates greater dispersion and consequently, a higher weight for that indicator. For this study, data reflecting positive feelings are recorded as matrix Z, a 1223 × 10 matrix, representing 10 indicators: mental toughness, goal encouragement, subjective thoughts, social resources, family relationship, social outlook, satisfaction in life, personality control, family income, and natural environment. Using the tool of information entropy, the weights of the indicators of positive factors were calculated to ensure the objectivity of the results as follows (21):

1. *Standardize data*:

$$Z_{\mathrm{ij}}=\frac{z_{\mathrm{ij}}-\min(z_{\cdot j})}{\max(z_{\cdot j})-\min(z_{\cdot j})},i=1,2\ldots 1223;j=1,2\ldots 10.$$

Where $z_{\mathrm{ij}}$ indicates i−th raw data corresponding to the j−th indicator, $z_{\cdot j}$ indicates the all data under j−th indicator.

2. *Calculate the probability matrix*: Calculate the proportion $p_{\mathrm{ij}}$ of the i *-* th data under the j−th indicator, regard it as the probability to be used in the calculation of information entropy, and get the probability matrix P. The formula is as follows:

$$p_{\mathrm{ij}}=\frac{z_{\mathrm{ij}}}{\sum_{k=1}^{n}z_{\mathrm{kj}}},n=1223;i=1,2\ldots n;j=1,2\ldots 10.$$

3. Calculate the information entropy of the j−th indicator:

$$E_{j}=-\frac{1}{\ln(n)}\sum_{i=1}^{n}p_{\mathrm{ij}}\ln(p_{\mathrm{ij}}),j=1,2\ldots 10;n=1223.$$

4. Calculate the discrimination of the j−th indicator:

$$F_{j}=1-E_{j},j=1,2\ldots 10.$$

5. *Calculate the weight*: the normalized discrimination is used as the weight of the corresponding indicator.

$$\omega_{j}=\frac{F_{j}}{\sum_{j=1}^{m}F_{j}},m=10;j=1,2\ldots 10$$

Through the above steps, we calculate the weights of 10 indicators in Table 4.

**Table 4:** Indicator weights for positive factors.

Indicator	Weight
Mental toughness	0.0512
Goal encouragement	0.0686
Subjective thoughts	0.0731
Social resources	0.0800
Family relationship	0.0426
Social outlook	0.0648
Satisfaction in life	0.1032
Personality control	0.1301
Family income	0.2639
Natural environment	0.1225

Analyzing these factors, it becomes evident that the criteria of familial financial stability, self-perceived personality, and the natural surroundings carry significant weight. This implies that seniors who find contentment in their household’s economic status, their personal character, and the natural ambiance that envelops them are more inclined to experience heightened happiness.

#### 4.3.2 Calculation of well-being scores for older people

Since each data value is one of 1, 2, 3, 4, 5, 6, there is no difference in order of magnitude and scale, so each older adult’s well-being score can be obtained by weighting the agreement score of these indicators:


$${\mathrm{df}}_{i}=\sum_{j=1}^{10}\omega_{j}z_{\mathrm{ij}},i=1,2\ldots 1223$$

Well-being scores portray the magnitude of the well-being of these older adults, with higher scores indicating greater well-being. Below we have calculated the mean scores for each of these indicators and for well-being, and the results are in Table 5.

**Table 5:** Mean scores for indicators and well-being.

Indicator	Mean score for each indicator	Mean score for well-being
Mental toughness	4.8365	
Goal encouragement	4.6623	
Subjective thoughts	4.7236	
Social resources	4.7367	
Family relationship	5.0720	4.4993
Social outlook	5.0016	
Satisfaction in life	4.6394	
Personality control	4.4652	
Family income	3.9477	
Natural environment	4.6206	

### 4.4 Modeling of negative factors based on confirmatory factor analysis and analytic hierarchy process

#### 4.4.1 Constructing the agreement evaluation structure of negative factors based on confirmatory factor analysis

First, KMO and Bartlett’s test of sphericity was conducted on the data of the 11 questions reflecting negative feelings in Table 6.

**Table 6:** Results of KMO and Bartlett’s test of sphericity.

Kaiser-Meyer-Olkin measure of sampling adequacy		0.866
Bartlett’s test of sphericity	Approx. Chi-Square	3769.752
	Degree of freedom	55
	Significance	0.000

KMO is a test used to assess the correlation and partial correlation between variables, the value is between 0 and 1, the closer the value is to 1, the stronger the correlation between variables, the weaker the partial correlation, the better the effect of the factor analysis. From our result, we had a KMO value of 0.866, which indicates that the source of agreement is suitable for the application of the factor analysis. Bartlett’s test of sphericity is used to test the independence of variables, and the significance is 0 (<0.05), which indicates that the sample is suitable for factor analysis (22).

**Figure 2:**
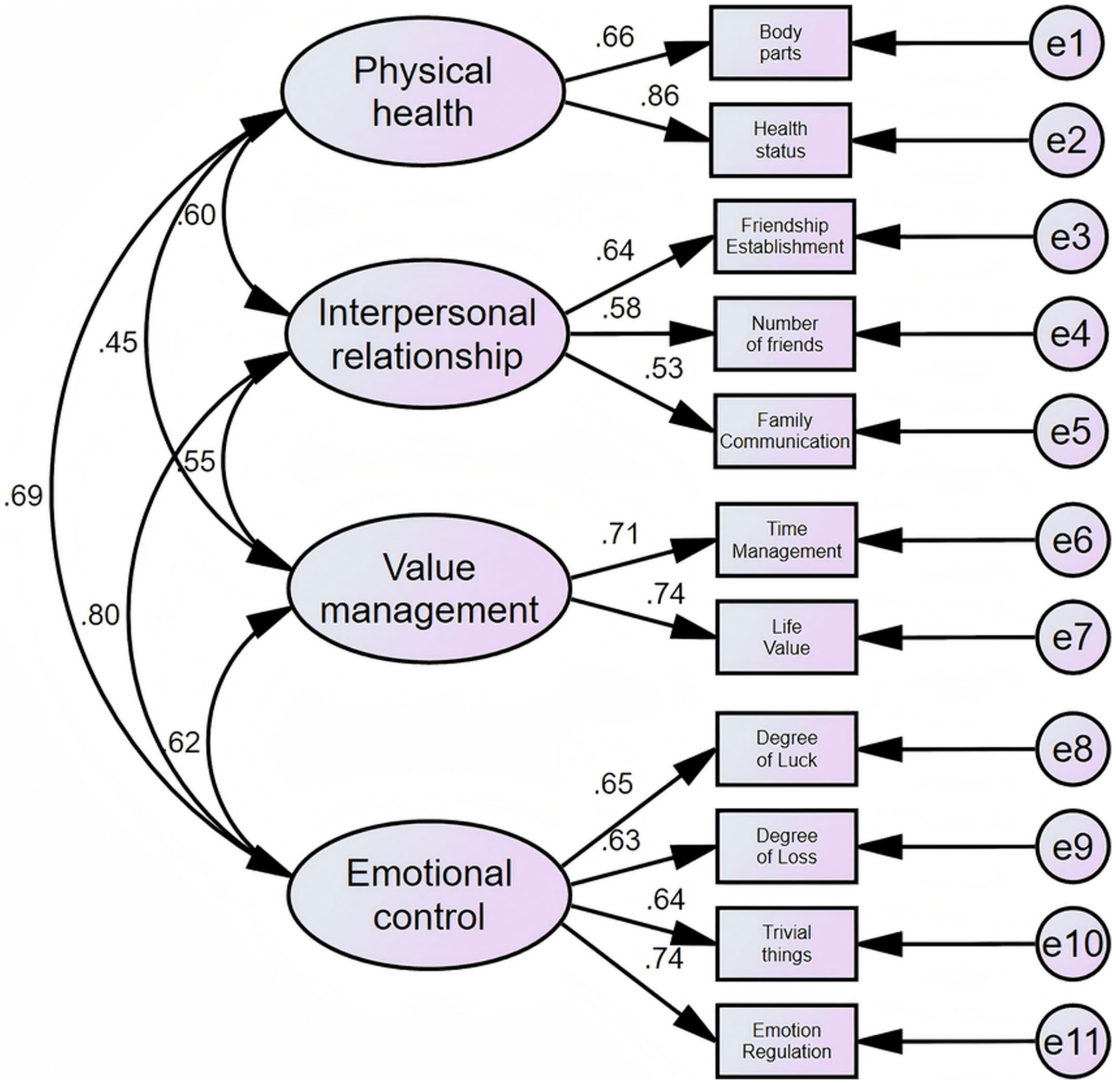
AMOS confirmatory factor analysis model diagram.

The path diagram and standardized path coefficients of the confirmatory factor analysis (23) are shown in Figure 2. Each question is a factor representing a tertiary indicator and is attributed to the corresponding secondary indicator.

Except for the coefficient value of physical health to value management (which is 0.45), which is less than 0.5, the coefficient value of each entry to the factor to which it belongs is greater than 0.5, which indicates that the classification is reasonable. The evaluation metrics for the overall fitting results of the model are given in Table 7.

**Table 7:** Fitting results for the CFA model.

Model metrics	Criterion for goodness of fit	Model performance	Model assessment
RMSEA	<0.08	0.062	Good
SRMR	<0.05	0.034	Good
CFI	>0.9	0.953	Good
TLI	>0.9	0.932	Good
IFI	>0.9	0.953	Good
GFI	>0.9	0.967	Good
AGFI	>0.9	0.943	Good
NFI	>0.9	0.943	Good
RFI	>0.9	0.918	Good

Each model metric meets the Criterion for goodness of fit, indicating that the structure of the agreement evaluation of negative factors is reasonable.

#### 4.4.2 Determining the weight of each indicator of negative factors based on analytic hierarchy process

Define the primary indicator of the agreement evaluation structure of the negative factors in 4.4.1 as the feeling of unhappiness, and take it as the goal layer of the negative factors, and the corresponding secondary and tertiary indicators as the criterion layer and the action layer, respectively.

Using the ratio scale of 1 to 9 and its inverse proposed by Saaty to establish a positive reciprocal matrix, the positive reciprocal matrix of the criterion layer to the goal layer of the negative factor (denoted as A) was obtained based on the table of ratio scale and the relative importance of each factor at corresponding level (24) by asking the professionals and referring to the relevant information as shown below:


$$A=[\begin{array}{cccc} 1 & 2 & 3 & 4\\ \frac{1}{2} & 1 & 2 & 3\\ \frac{1}{3} & \frac{1}{2} & 1 & 2\\ \frac{1}{4} & \frac{1}{3} & \frac{1}{2} & 1 \end{array}]$$

After calculation, the maximum eigenvalue of matrix A: λ=4.030983 , and its normalized eigenvector is:


$$\omega ={\left(\omega_{1},\omega_{2},\omega_{3},\omega_{4}\right)}^{T}$$

$$\omega ={\left(0.4673,0.2772,0.1601,0.0954\right)}^{T}$$

Consistence Index $\mathrm{CI}=\frac{\lambda -n}{n-1}=\frac{4.030983-4}{4-1}=0.010328$ , the random index is 0.9 (when n = 4) given by Saaty, the consistency ratio is obtained: $\mathrm{CR}=\frac{\mathrm{CI}}{\mathrm{RI}}=\frac{0.010328}{0.9}=0.011475<0.1$ , so matrix A passes the consistency test, and the weight of the criterion layer relative to the goal layer is obtained:


$$W_{A}={\left(0.4673,0.1601,0.2772,0.0954\right)}^{T}$$

Similarly, by asking the professionals and referring to the relevant information, we get the positive reciprocal matrices of the action layer of negative factors to the criterion layer (physical health, interpersonal relationship, value management, and emotional control in order) are noted as A1, A2, A3, A4, and there are:


$$A1=[\begin{array}{cc} 1 & \frac{1}{2}\\ 2 & 1 \end{array}];A2=[\begin{array}{ccc} 1 & 2 & \frac{1}{3}\\ \frac{1}{2} & 1 & \frac{1}{5}\\ 3 & 5 & 1 \end{array}]$$

$$A3=[\begin{array}{cc} 1 & \frac{1}{2}\\ 2 & 1 \end{array}];A4=[\begin{array}{cccc} 1 & \frac{1}{2} & \frac{1}{3} & \frac{1}{4}\\ 2 & 1 & \frac{1}{2} & \frac{1}{3}\\ 3 & 2 & 1 & \frac{1}{2}\\ 4 & 3 & 2 & 1 \end{array}]$$

In particular, for 2nd-order matrices, the consistency ratio does not apply, and it is usually not necessary to perform consistency tests on 2nd order matrices because they are inherently consistent. The indicators corresponding to these 4 matrices are obtained as shown in Table 8.

**Table 8:** Calculation results.

Matrix	Maximum eigenvalue	Eigenvector (after normalization)	CI	RI	CR	Accept or not
A1	2.0000	(0.3333, 0.6667)	0	0	\	Yes
A2	3.0037	(0.2297, 0.1220, 0.6483)	0.0018	0.58	0.0032	Yes
A3	2.0000	(0.3333, 0.6667)	0	0	\	Yes
A4	4.0310	(0.0954, 0.1601, 0.2772, 0.4673)	0.0103	0.90	0.0115	Yes

The results showed that all four matrices pass the consistency test. Furthermore, the consistency ratio of hierarchical total sorting of negative factors was obtained: ${\mathrm{CR}}_{A}=\frac{\sum_{j=1}^{4}{\mathrm{CI}}_{j}W_{A,j}}{\sum_{j=1}^{4}{\mathrm{RI}}_{j}W_{A,j}}=0.006<0.1$ , it had a more satisfactory consistency and the results of this analysis were accepted.

#### 4.4.3 Calculation of unhappiness scores of older people

An evaluation system of the sources of unhappiness feelings of older adults was established and the results are shown in Table 9.

**Table 9:** Evaluation system of sources of feelings of unhappiness.

Goal layer O	Criterion layer $y_{i}\;$ and its weight in relation to goal layer $b_{i}$	Action layer $x_{\mathrm{ij}}$ and its weight in relation to criterion layer $a_{\mathrm{ij}}$	Action layer’s weight in relation to goal layer $b_{i}*a_{\mathrm{ij}}$
Older people’s feelings of unhappiness	Physical health (0.4673)	Body parts (0.3333)	0.1558
		Health status (0.6667)	0.3115
	Interpersonal relationship (0.2772)	Friendship establishment (0.2297)	0.0637
		Number of friends (0.1220)	0.0338
		Family communication (0.6483)	0.1797
	Value management (0.1601)	Time management (0.3333)	0.0534
		Life value (0.6667)	0.1067
	Emotional control (0.0954)	Degree of luck (0.0954)	0.0091
		Degree of loss (0.1601)	0.0153
		Trivial things (0.2772)	0.0264
		Emotion regulation (0.4673)	0.0446
Unhappiness score	$\mathrm{DF}=\sum_{i=1}^{4}\sum_{j}b_{i}a_{\mathrm{ij}}x_{\mathrm{ij}}$		

Upon examination, it is discerned that the parameters of health status, familial interaction, and physical well-being are accorded greater importance. This suggests that older adults who harbor concerns regarding their health, encounter barriers in communicating with their kin, and frequently experience discomfort are predisposed to a state of increased unhappiness.

Accordingly, the scores for each older adult’s criterion and goal layers can be derived from their action layer scores, with the unhappiness score reflecting the extent of their unhappiness—higher scores denote greater unhappiness. Below, we have calculated the average scores for each of these indicators and unhappiness, with the results in Table 10.

**Table 10:** Scores for each indicator of the unhappiness scale.

The factors of action layer	The mean scores of action layer	The indicators of criterion layer	The mean scores of criterion layer	Unhappiness score
Body parts	3.5045	Physical health	3.2368	3.0620
Health status	3.1030			
Friendship establishment	2.6697	Interpersonal relationship	2.9286	
Number of friends	3.2535			
Family communication	2.9591			
Time management	2.8545	Value management	2.8441	
Life value	2.8389			
Degree of luck	2.9509	Emotional control	2.9587	
Degree of loss	2.8659			
Small things	3.0335			
Emotion regulation	2.9477			

As noted in section 4.3.2, the mean well-being score is 4.499, situated between 4 and 5. This suggests that older individuals, on the whole, fall between somewhat agreeing and agreeing on these positive aspects. Conversely, the mean unhappiness score and the corresponding criterion layer scores approximate 3, indicating that older adults generally lean toward somewhat disagreeing on these negative aspects. Thus, the overall well-being of older adults can be regarded as robust.

### 4.5 The role of different socioeconomic status on overall subjective well-being scores

We have established an evaluative framework for gauging the happiness and unhappiness of older adults, deriving an overall happiness score Z(=df−DF) by subtracting the unhappiness score from the happiness score. A higher score indicates greater overall happiness and, to a significant extent, suggests psychological well-being. However, further detailed analysis is warranted to discern the nuances in the overall happiness of older adults and to formulate more precise recommendations.

To this end, we initially determine whether significant disparities exist in the overall happiness scores among older adults with varying classification outcomes using ANOVA. Subsequently, we integrate these findings with the satisfaction evaluation system to propose targeted recommendations for enhancing the overall happiness of older adults.

**Figure 3:**
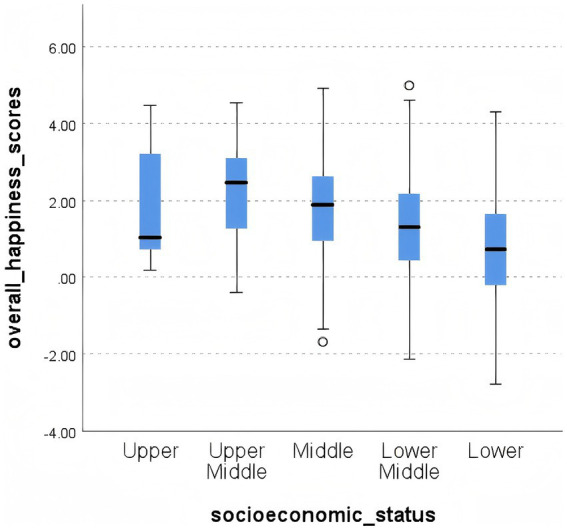
Box plots of overall happiness scores by socioeconomic status.

The dataset from question A43e and the calculated overall happiness scores were imported into SPSS, confirming normal distribution via KS (Kolmogorov–Smirnov Test) (25) and SW tests. It was determined that group variances were homogeneous (with chi-square test significance above 0.05), and group means were significantly different (ANOVA test significance below 0.05). It was concluded that older adults with varying socioeconomic statuses have distinct overall happiness scores. The means and box plots of the overall happiness scores for each group are in Table 11 and Figure 3 (26).

**Table 11:** Mean of overall well-being scores by socioeconomic status.

Targets	Numbers	Average value
Upper	10	1.7750
Upper-middle	74	2.2541
Middle	455	1.8355
Lower-middle	370	1.3428
Lower	314	0.7679
Total	1,223	1.4371

This suggests that older adults in lower socioeconomic strata exhibit lower overall happiness scores and are more susceptible to psychological issues, thus necessitating additional attention. U. S. - based research demonstrates that low—income older adults frequently encounter diminished quality of life and heightened psychological distress due to economic pressures, a finding that aligns with the circumstances of this group in China and underscores the urgency of focused attention. Additionally, we observe that the overall happiness of the upper and middle strata is comparable, with the upper—middle stratum displaying the highest overall happiness. The “diminishing marginal effect of income” theory proposed by Western research posits that as economic income levels rise, the enhancement effect of income on happiness gradually diminishes. Beyond a certain threshold, further income increases have limited impact on happiness enhancement and may even lead to a decline (27). This relationship is also evident in the Chinese context, where the upper - middle class, benefiting from economic stability and greater opportunities for social engagement, achieves a balance between economic security and social participation, thereby enjoying the highest level of happiness. Notably, research from Nordic countries like Sweden and Norway reveals that a robust social welfare system (e.g., free medical care, substantial old—age allowances) can effectively mitigate the impact of economic status disparities on well-being. This highlights the pivotal role of social policy in regulating the relationship between economic status and well-being, offering significant international insights for optimizing future policies in China to enhance the overall well-being of older adults across diverse socioeconomic strata.

## 5 Conclusions and recommendations

### 5.1 Conclusions

#### 5.1.1 Positive factors have a positive effect on the subjective well-being of older adults

The positive factor model, as outlined by the information entropy method and systematic cluster analysis, highlights the beneficial effects of positive factors such as family income, personality control, and natural environment on the subjective well-being of older adults. These factors enhance life satisfaction and emotional states, thereby improving the overall quality of life. They foster social engagement and a sense of belonging, which in turn enhance well-being. Moreover, these positive factors act as psychological capital, equipping older individuals to navigate life’s challenges and mitigate negative emotions more effectively.

#### 5.1.2 Negative factors have a negative effect on the subjective well-being of older adults

The negative factor model, established through confirmatory factor analysis and analytic hierarchy process, reveals the substantial negative impact of factors such as health status, family communication, and body parts on the subjective well-being of older adults. Collectively, these factors impair mental health and life quality by affecting daily functioning and self-care, increasing loneliness and social isolation, diminishing life purpose and meaning, and destabilizing emotions, thereby reducing subjective well-being. The compound effects of physical health issues leading to restricted mobility and social engagement, loneliness from deficient interpersonal connections, a lack of purpose due to poor value management, and emotional instability from inadequate emotional control all contribute to a decline in older individuals’ emotional states and life satisfaction, culminating in a reduced sense of well-being.

The findings of this study provide a new perspective for understanding the complexity of subjective well-being in older adults and provide a scientific basis for developing strategies to enhance their mental health and well-being. Through targeted interventions, the quality of life and well-being of older adults can be effectively improved, and social harmony and stability can be promoted.

### 5.2 Recommendations

1. *Financial stability for older adults*: it is imperative for governments to implement tax incentives and social security disbursements to safeguard the economic well-being of senior citizens. Additionally, non-profit entities should offer comprehensive financial literacy programs aimed at assisting the aged in astutely managing their retirement funds. This proactive approach not only mitigates financial strain but also bolsters their psychological comfort and overall sense of security.
2. *Cultivating personality and self-insight*: community mental health hubs should routinely convene workshops focused on the refinement of personal character and the enhancement of self-awareness. These initiatives should be designed to encourage seniors to delve into their individual passions and principles, thereby amplifying their self-efficacy and fostering a constructive evolution of their personal identities.
3. *Integration with nature*: municipal authorities ought to expand the allocation of verdant areas and actively engage older adults in horticultural and naturalistic pursuits. Immersion in natural settings is instrumental in alleviating stress, thereby augmenting the psychological well-being and elevating the life quality of senior individuals.
4. *Health surveillance and wellness*: medical establishments must offer periodic complimentary health assessments and curate detailed health profiles for the senior population. This enables the proactive identification and management of health issues. A robust physical constitution underpins mental health, and consistent health surveillance is pivotal for older adults to sustain an optimistic mental disposition.
5. *Intergenerational dialogue and affective bonding*: it is essential for families to foster open lines of communication across generations and to orchestrate frequent familial reunions. Such efforts fortify the emotional fabric that unites family members. Robust familial interactions serve as a conduit for emotional succor, mitigating the sense of isolation and fostering a robust social network for older adults.
6. *Enhancing physical vigor*: the realm of sports and wellness must advocate for exercises tailored to the senior demographic, including practices like Tai Chi and yoga, with an emphasis on holistic bodily health. Through regular physical engagement, older adults can augment their fitness levels, diminish disease susceptibilities, and concurrently elevate their self-assurance and psychological well-being.

## Data Availability

The original contributions presented in the study are included in the article/supplementary material, further inquiries can be directed to the corresponding author.
